# Co-designing ‘gene’, a smartphone app for genetics education and empowerment with and for the British Pakistani community: a methodological summary of the GENE-Ed project

**DOI:** 10.1007/s12687-025-00789-0

**Published:** 2025-04-12

**Authors:** Norina Gasteiger, Alan Davies, Nasaim Khan, Amy Vercell, Dawn Dowding, Syed Mustafa Ali, Angela C. Davies

**Affiliations:** 1 Division of Nursing, Midwifery and Social Work, School of Health Sciences, The University of Manchester, Manchester, UK; 2 NIHR Applied Research Collaboration, Greater Manchester, UK; 3 Division of Informatics, Imaging & Data Sciences, School of Health Sciences, The University of Manchester, Manchester, UK; 4 The Christie NHS Foundation Trust, Manchester, UK

**Keywords:** Co-design, Genetics, Consanguinity, British Pakistani, Genetic literacy, Co-creation

## Abstract

**Introduction:**

A lack of culturally appropriate genetic information prevents the British Pakistani community from engaging with genetic services. The GENE-Ed project focussed on the development of an educational app with and for the Pakistani community. A secondary aim was understanding how to engage the community in research.

**Methods:**

We used an iterative co-design and co-creation approach including four phases to develop the Gene app. Phase 1 included seven interviews with community members to explore genetics understanding and define the requirements. Phase 2 included reviewing smartphone apps and research on digital patient-facing interventions for genetics understanding. Phase 3 included developing the app and obtaining initial feedback. In Phase 4, feedback was obtained from five community members using the System Usability Scale (SUS), a bespoke survey and observations.

**Results:**

Four themes were identified in the interviews: current awareness of genetics; consanguinity, religion and cultural influence; presenting genetics information in a new digital resource and dissemination; information-sharing and uptake. The reviews highlighted an absence of culturally sensitive, accessible and evidence-based digital resources. Initial feedback included altering the animations and images within the app and simplifying the text. The mean SUS score was 87, indicating excellent usability. The written information, animations and videos were acceptable to participants, and they tended to trust the information in the app. During feedback, community members responded well to different methods but struggled with written open-ended survey questions.

**Conclusion:**

The co-design approach was essential to developing an acceptable resource for the British Pakistani community. Future clinical testing is needed.

**Supplementary Information:**

The online version contains supplementary material available at 10.1007/s12687-025-00789-0.

## Introduction

Genetic variations can lead to inherited disorders when they are passed down through generations. It has been estimated that around 30,000 children in the United Kingdom (UK) are diagnosed with a genetic condition annually (Gene People 2020). However, rates are higher among populations such as the Pakistani community, which practices consanguineous (close relative) marriage (Merten 2019; Temaj et al. 2022; Verma and Puri 2015). According to (Sheridan et al. 2013), 31% of anomalies in the Pakistani community may be attributed to consanguinity. Additionally, 6.2% of births from first-cousin couples have been found to have congenital anomalies, compared to 2.3% of births from unrelated White British couples (Sheridan et al. 2013). Impacts can be significant, causing infant mortality and morbidity (Salway et al. 2016), feelings of guilt, depression, anger and anxiety (McAllister et al. 2007) and increased healthcare costs to families who provide care, and the healthcare system. For example, healthcare expenditure for managing the genetic condition β-thalassemia major over 50 years is around £483,454 per patient (Weidlich et al. 2016).

Affected or at-risk individuals or their family members may seek to engage with a genetic counselling service, which may involve undergoing carrier testing (also known as carrier screening) in which a blood test is conducted to determine whether an individual is a carrier of certain genetic conditions. However, literature has pointed to a lack of awareness, poor genetic literacy and barriers to accessing genetic services among British Pakistani communities (Ajaz et al. 2015; Shaw and Hurst 2008). Participants have previously discussed a need for a culturally competent service by people who understand the importance of cousin marriages to the community (Ajaz et al. 2015). Research has also highlighted that the cascading of information among family members in communities where harmful gene changes have been identified is often poor, so multiple affected births (and deaths) ensue (Salway et al. 2016; Khan et al. 2011; Khan et al. 2016). Nevertheless, to our knowledge, no research has specifically explored what the community want regarding information on genetics or genetic counselling services.

In the UK, several strategies and guidelines have highlighted the need for clear, culturally acceptable and equitable access to services. For example, the NHS England Genomics Strategy emphasises equitable access to services co-designed with the communities they serve (NHS England 2022). Other guidance has highlighted the need for NHS hospital trusts and community providers to meet their patients’ communication needs by providing accessible information, including in their preferred languages (National Institute for Health and Care Excellence Antenatal care 2022). Despite this guidance, there is no culturally sensitive and little information overall on genetic implications for consanguineous marriages on NHS websites. It has also been reported that digital resources specifically aimed at genetic education outside of the NHS context are few, with a paucity of these resources created directly by a reliable or credible agency (Talwar et al. 2019). This unmet need is contributing to increasing inequities in accessing and benefitting from genetics services for the British Pakistani community. It also highlights a need to determine the requirements for and develop a new appropriate resource, with and for the community.

In 2021 we collaborated with an NHS genetic counsellor, Naz Khan (NK) with 20 years of clinical and service development experience working with the Pakistani community in Northwest England to begin our work on the GENE-Ed project. It was clear that there was a significant need to co-create a culturally sensitive and accessible information resource for at-risk British Pakistani communities. The aim of the GENE-Ed project was to develop a new resource with and for the Pakistani community to help distribute accessible genetic information and empower families to make informed decisions about childbearing, genetic counselling and carrier testing. We were also interested in understanding how to best engage the community in research, as they are often underserved (Ali et al. 2024). Reasons for the exclusion of ethnic minority groups from research have pointed to negative attitudes and preconceptions such as poor English language skills, inadequate transport or not being able to keep appointments or comply with the research protocol (Redwood and Gill 2013; Lo and Garan 2008).

## Methods

### Co-design approach and collaborators

**Fig. 1:**
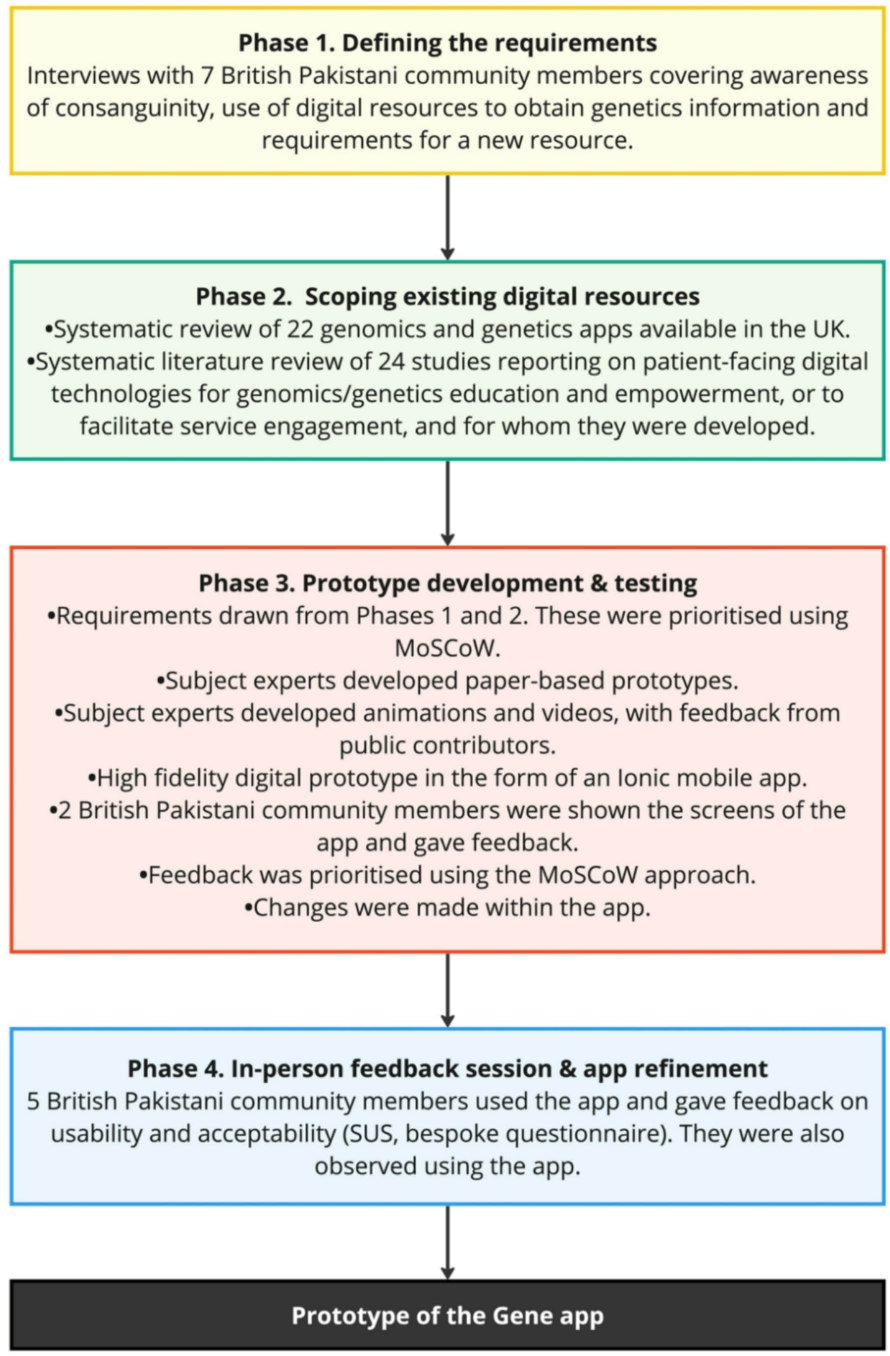
Diagram showing the four key phases of the GENE-Ed project

The GENE-Ed project used a co-design approach, whereby the expertise of end users (stakeholders) was drawn on, and they were involved in designing the product from the idea generation stage (Sanders and Stappers 2008). Co-design is a democratic approach that actively involves stakeholders in the creation process and emphasises participation, iterative development, collective ownership, and a practical focus on achieving specific outcomes and intents (Carr et al. 2009; Donetto et al. 2014). An iterative co-design approach similar to that recommended by (Davies et al. 2020) was followed, involving key stakeholders in each stage of design and development and the iterative production of prototypes. This approach was deemed most appropriate given the lack of evidence on what the British Pakistani community want in a genetics information resource, and to ensure that the final product was acceptable to the community. There were four key phases of the project with the last two phases dedicated to the development and refinement of the app (see Fig. 1).

The team includes digital health researchers at The University of Manchester with experience in digital health inequity, co-design, clinical genetics service and evaluation research. Two are members of the South Asian community in Greater Manchester. Our collaborators included Blackburn with Darwen Borough Council and four Pakistani public contributors who have experience in accessing genetics services. Blackburn with Darwen Borough Council is the local authority for Blackburn with Darwen, a unitary authority in Lancashire, England. The council is responsible for providing most local government services for the area. Given high rates of child deaths from genetic disorders in this area, a Community Genetics Service was commissioned by the borough’s public health team. The overall aim of the service was to improve awareness of genetics amongst the community and professionals and ensure that affected families receive the correct specialist support. This was done via a network of champions who supported families by enquiring whether necessary referrals had been made and if information received at the appointment had been understood. Representatives from the council attended the GENE-Ed project meetings and gave input on the progress and approach taken.

### Recruitment and ethics

We first tried to recruit community participants using posters in various community and primary care settings. However, response was limited as ‘traditional’ approaches can be unsuccessful due to issues of trust relating to external researchers. Instead, NK contacted and communicated with potential participants in person via known community groups. This grassroots approach generated a better response due to established strong working relationships with the community organisations established over time through NK’s public health outreach work.

All participating community members were aged over 18 years and able to read, write and speak in English, though literacy was varied. Potential participants registered their interest with NK, who outlined the information sheet with them over the telephone and answered any questions. Everyone was compensated with a £30 voucher. The University of Manchester’s Proportionate University Research Ethics Committee approved each phase of the project (Reference 2020-10290-16637).

### Phase 1. Defining the requirements

The first phase was dedicated to understanding how consanguinity and genetics are understood within the community and how to best disseminate culturally sensitive information. AD, ACD and NK conducted seven in-depth interviews with community members, online using Zoom. The semi-structured interview schedule covered understanding and awareness of consanguinity and genetics in the community, digital resources to obtain genetics information and the requirements for a new resource (see Appendix 1 for interview questions). Interviews ranged from 27 to 56 min (average 42.6 min) and were audio-recorded and transcribed by a university-approved service.

The data were analysed thematically (Braun and Clarke 2017) using NVivo 12. NG and SMA first familiarised themselves with the data. They then independently coded one transcript and discussed their interpretations. NG then coded three other transcripts, with SMA checking the codes and identifying any discrepancies in agreement. The remaining transcripts were then coded and themes were identified, along with supporting quotes. The wider team reviewed the themes, checking they were meaningful.

The participants were six females and one male. Four identified as Pakistani, and three as British Pakistani. Their ages ranged from 24 to 56 (average: 37.6 years). Four were employed at the time of the interview, and education levels ranged from foundation entry-level and diplomas to master’s degrees. Three had previously been a patient in genetics services, three had not and one was a parent to a child patient.

### Phase 2. Scoping existing digital resources

To minimise any potential for duplication and digital waste, we wanted to ensure that there were no existing and appropriate (high-quality) technologies targeted at the community. We therefore conducted two pieces of work- a systematic review of apps and a systematic literature review of published studies. Both have been published (Gasteiger et al. 2022; Gasteiger et al. 2023) and should be referred to for more detail.

First, we conducted a systematic review of the content, functionality, and quality of patient-facing genetic and genomic mobile apps available in the UK. Apps were downloaded for evaluation using the Mobile App Rating Scale (Stoyanov et al. 2015), Flesch-Kincaid readability metrics (Flesch 1979; Kincaid et al. 1975) and the IMS Institute for Healthcare Informatics functionality score (Aitken et al. 2013).

In our systematic review of digital interventions for genomics and genetic education, empowerment, and service engagement, we explored existing patient-facing digital technologies (Gasteiger et al. 2023). Literature was retrieved from eight databases and quality was assessed using the Mixed Methods Appraisal Tool (Hong et al. 2018). The review adhered to the Preferred Reporting Items for Systematic reviews and Meta-Analyses guidelines (Page et al. 2021).

### Phase 3. Prototype development and testing

The requirements for the app were drawn from Phases 1 and 2 and were broken down into themes. These were then prioritised by the app development team according to the MoSCoW strategy (Clegg and Barker 1994). Requirements that were rated as ‘must have’ or ‘should have’ were prioritised to generate a prototype of the app.

The app was developed as a cross-platform app to make it easier to deploy across the Android and iOS platforms with minimal additional development time. To achieve this, the Ionic Angular framework (v6) (Ionic 2024) was used to code the application with Capacitor (a native runtime for PWA, iOS/Android cross-platform and web-native apps). An iterative approach to app development was applied using the Agile Scrum framework and Kanban, as recommended by (Davies and Mueller 2020). First, initial paper prototypes were developed through a series of workshops with subject matter experts during the COVID-19 pandemic.

Secondly, the group used a paper prototyping method, using mocked up phone screens to explore how the content might be displayed, which was then discussed within the group, to agree preferred formats. The group then explored how content would be conveyed including text, animations and videos. Animations and a high-fidelity digital prototype in the form of an Ionic mobile app were then developed, drawing on feedback from two community members. Lastly, two female participants from the original seven took part remotely in an online review of the app and its content in early September 2023, which was recorded using Zoom. The app was presented in a browser window using one of Google Chrome’s inbuilt phone frames (Nexus 5 × 412 × 732) available via the developer tools menu. The screens of the app were presented one by one to the participants and then questions from the app evaluation survey (Appendix 2) were used to elicit feedback and comments from participants.

One of the participants had previous experience of accessing genetic counselling, the other did not. Proposed changes or issues were documented during the session. Following the session, the issues and proposed changes were presented to the rest of the project team and discussed.

### Phase 4. In-person feedback session and app refinement

To test whether the app was usable and acceptable by the community, we conducted a mixed-methods evaluation of the Gene app prototype in November 2023 with five new participants recruited from the Pakistani community (by NK) conducted over two hours. Only the English version of the app was evaluated due to limitations of the spoken languages of the project team able to lead the feedback session. The session was held at a family hub in Blackburn, which was convenient for all participants to access. We aimed to recruit five participants, which has been recommended for being able to identify 80% of a product’s usability issues (Lewis 1994; Virzi 1992). Additionally, this number was perceived as being feasible to observe at one time.

#### Procedures and measures

The project and session were introduced by two researchers (ACD and NG), and written consent was then obtained. Participants were each given a smartphone (Samsung Galaxy S6) which hosted the Gene app and were asked to use the app for 30 min. They were also given headphones to minimise any disruptions to one another whilst listening to videos.

Participants then completed surveys with questions about their demographics (age, gender and ethnicity) and whether they had accessed a genetics service (yes; no). Nine Likert questions with five response options (ranging from strongly disagree to strongly agree) asked about the acceptability of the app. Questions covered the written information, animations and videos and layout of the content in the app, navigation and usability and trust in the information. Open-ended questions also asked for justification for their responses, ideas for improvement and additional feedback. Appendix 2 presents the survey questions. Data were analysed descriptively on Microsoft Excel using means and standard deviations.

Lastly, the 10-item System Usability Scale (SUS) was administered (Brooke 1996), as shown in Appendix 2. The SUS data was analysed on IBM SPSS (version 27), consistent with previous approaches (Dowding 2019; Gasteiger 2024). Responses were converted to range from 0 to 4 (the higher score indicating the most positive response), summed and multiplied by 2.5. This gave a score ranging from 0 to 100. As interpreted by Bangor et al. (Bangor et al. 2009), a score below 50 indicates poor usability, over 70 indicates good usability and over 85 reflects excellent usability.

Researchers (NG and ACD) also made observations and fieldnotes throughout the session, noting discussion topics between the participants, how the app was used, any verbal feedback given, challenges experienced in using the app and responses to the data collection methods (e.g., whether any clarification or support to participate was requested). They also supported the participants in responding to the open-ended questions by offering to transcribe their verbal responses verbatim if there were any issues regarding English literacy.

#### Participants and analysis

All five participants identified as female, three as Pakistani and two as Indian. The mean age was 43.2 years (range 35–62). Three reported having a prior interaction with the genetic service, while two had no interaction.

Qualitative data was analysed using a content synthesis approach to identify certain ideas and inter-relate or categorise them [41]. One researcher (NG) first analysed the data by adding, changing and moving codes, determining key categories/themes and selecting quotes to best illustrate them. A second researcher (ACD) then reviewed the analysis for consistency in interpretation. The findings were also discussed during a team meeting.

## Results

### Phase 1. Defining the requirements

Four themes were identified across the interviews: (1) current awareness of genetics, (2) consanguinity, religion and cultural influence, (3) presenting genetics information in a new digital resource and (4) dissemination, information-sharing and uptake.

#### Theme 1. Current awareness of genetics

When explaining genetics, participants identified traits that their children had inherited from one parent (e.g., being flat-footed, wearing glasses or hearing loss). When questioned regarding how to explain genetics to family members, participants suggested making models out of Play-Doh or using diagrams or analogies related to cooking, prayer beads, building and making a cup of tea.

*Like making a cup of tea*,* the slight variation of the milk… it makes a difference.*

It was emphasised that it’s not about how the information is relayed but who is presenting the information that is important, as community members are more likely to trust people they know and respect.

*I think it’s a cultural issue…I think people are more likely to trust someone that they know in their own community.*

Participants were reflective of how they would explain genetics to elders in the community, suggesting that they play an important role in medical decision-making. In this regard, multiple interrelated barriers to explaining genetics were identified, including language, education, and health literacy and that many words (e.g., genes) do not directly translate to Urdu nor are commonly understood.

*From a Pakistani community*,* our elders don’t always understand the genetics condition. They just think it’s a****bimari****and****bimari****just means an illness that will go away or will be treated with a bit of paracetamol.*

#### Theme 2. Consanguinity, religion and cultural influence

Only one participant was aware of consanguinity as a terminology. However, everyone was aware of cousin marriages, although there were many different interpretations. These ranged from cousin marriages being directly associated with arranged marriages, being only limited to first or second cousins or any extended relative of the *“same blood.”*

Participants had some level of understanding about cousin marriages, but most of them had different interpretations, which were necessarily not correct.

*….everybody said*,* oh*,* what rubbish she’s talking*,* like cousin marriage has been happening since our Prophet’s time*,* it’s not a valid reason to break an engagement with your uncle’s son*,* like it’s a family thing*,* you can’t do that on just a very silly assumption that this can happen.*

As participants described, women are perceived to be responsible for passing down a genetic condition in their community. Therefore, it reduces women’s marriage prospects hugely. One of the participants described how:

*In a community where girls are being rejected on the basis of their colour and their height*,* bringing this into the picture will make it more difficult*,* I think*,* where you are rejecting people on the basis of*,* okay*,* they have got this kind of disease in the family so it’s a big no.*

#### Theme 3. Presenting genetics information in a new digital resource

Participants had requests related to the written information, language, including engaging visuals and embedding trust (using NHS branding) and featuring patient stories (see Table 1).

**Table 1:** Requirements for the new app presented as sub-themes with supporting quotes (presented in italics)

Sub-theme	Participant requests and quotes
Written information	Content • Written information needs to be concise, free of jargon and simple. • They requested information on the risk of genetic disorders, how close relative marriages increase risks for having an affected child, the impact of genetic disorders (e.g., how life-threatening and life-changing they are), which disorders are commonly tested for, how to get a genetics test or referral to a genetics counsellor and what happens during these appointments. • Contact details to speak to a volunteer service, healthcare professional or other people with the same genetic condition could be added. • The app should not contain information related to religion as it may deter older community members. *I think the elder generation*,* in particular*,* would probably stay away from the app saying that*,* you know*,* they’re just saying don’t marry your cousin*,* more than the genetic information side.* Design • Information should be organised by tabs, so users can find the information easily. • Active links could direct users to further information (e.g., on specific genetic conditions).
Language	● Information should be presented in Urdu and English. Many of the younger generation are losing their Urdu, but the older generation speaks Urdu most confidently. *It definitely needs to be bilingual*,* so it caters for everyone.*
Engaging visuals and embedding trust	Content ● Visual representations (e.g., a cartoon or a video) may reassure users that the genetic counsellor can be trusted. ● The new resource should feature interactive elements, such as personalisation of content, being able to select things, quizzes, searching for content or inputting personal details to receive advice on what to do next (e.g., to see a GP if there is a high risk that a genetic disorder runs in the family). As reflected by one participant, interactivity is important for ongoing use: *You have to be able to click on things*,* and get to somewhere*,* than just reading a load block of information.* Design • The app should be visually engaging, using a multitude of colours, icons, infographics, pictures and short videos. • Participants likened the NHS to being trustworthy. Using NHS branding (blue colour and logo) can elevate the credibility of the app. *I would trust that*,* if there was anything with an NHS logo on it.* • The icon needs to be obscure and unrelated to genetics, so users could hide using it, particularly if sharing their phone with family.
Patient stories	● Some thought that images of small children with severe disabilities can be upsetting and might discourage people from engaging with the app. ● Others thought that parents sharing their stories on how they engaged with genetic services can be empowering, as users can learn about the support available and be reassured that their child’s genetic disorder is not their fault. ● Stories may be comforting to older adults in the community and help them understand the problem.

#### Theme 4. Dissemination, sharing and uptake of genetics-related information

Participants discussed that linking the app with existing services and community groups would widen uptake, as people are unlikely to think about genetic conditions until they are affected. A participant explained that much more marketing would need to happen if the app was standalone, given that there are *“millions of apps”* on the app stores.

There were many suggestions for how the app may be disseminated to young people, including sharing it on Facebook groups, YouTube and Instagram, using a distinct hashtag: *If you’re really trying to get the younger generation*,* you might start a campaign*,* a hashtag*,* and try to promote it with maybe famous bloggers*,* things like that.*

To engage the community more broadly, participants suggested using influencers and high-profile community members to promote the app and also sharing it on community social media groups (e.g., from mosques), at bus stops and in local children’s centres and on marriage or Muslim dating websites. Healthcare workers and settings were also identified as potential avenues for dissemination. For example, via midwives, distributing leaflets at GP clinics, on the NHS website or placing posters in waiting areas of hospitals and pharmacies.

It was emphasised that in-person communication and word-of-mouth were extremely important in the community. This was also important for people who do not speak English confidently or who may not respond to leaflets. Participants suggested that community events (e.g., health seminars) or engaging people while waiting at the doctor’s surgery could be an appropriate way to engage the community and answer any questions.

*I strongly believe that it has to be face-to-face communication in terms of maybe health seminars*,* awareness events that are going on within the local community. I don’t think it’s something that if it was posted through the door or a leaflet*,* that they would actually take the time to sit down and read it.*

Participants also explained that despite initially using the app alone, they would share the information with their partners and family members, especially if it was serious (e.g., recommended seeing a GP). One participant explained that information-sharing in families was normal due to the fact that many people *“don’t have the privacy of their own phone.”* Others explained that they simply like to share relevant information.

*I’d probably use it myself first. I’d get to see the information. And then I would pick my targets*,* which I normally do*,* would be my sister-in-laws*,* who have got children of marriageable age*,* and then I’d be saying*,* have you seen this*,* I’ll send you the link*,* why don’t you download it*,* have a look in your own time. Or I’d say*,* you know*,* click on that*,* and just see that video about that family and what happened to them and how it impacted and the things they had to go through and…we might look at it together.*

### Phase 2. Scoping existing digital resources

In the systematic review of the content, functionality, and quality of patient-facing genetic and genomic mobile apps available in the UK we identified 22 relevant apps that were aimed at educating users [24]. There were many more apps available from the two UK app stores (*n* = 731), but the two stages of screening highlighted that most did not meet the eligibility criteria for myriad reasons, including those that relied on DNA sampling, not being patient-facing, not focussing on genetic conditions, or not being in English. Of the 22 apps that were reviewed, half did not meet the minimum acceptability criteria (quality), only three were affiliated with a registered charity or healthcare body, and none had been formally trialled or tested. This emphasises the need for a culturally sensitive, accessible, evidence-based app to be developed aimed at improving genetic literacy within specific communities.

In our systematic review of digital interventions for genomics and genetic education, empowerment, and service engagement, we explored existing patient-facing digital technologies [25]. Twenty-four studies were included, of which twenty-one were deemed moderate or high quality. Most (88%) were carried out in America, the majority (79%) were conducted within a clinical setting, more than half (63%) of the interventions were web-based tools, and almost all (92%) focussed on educating users. However, few of the studies were aimed at improving the empowerment of patients or were community-based, and none targeted the Pakistani community, thus highlighting the need for future work to focus on co-developing content with end users.

### Phase 3. Prototype development and testing

Themes presenting the requirements for the app included features, accessibility, design, content, quality and security. These were prioritised by the app development team (Table 2).

**Table 2:** Prioritisation of captured requirements using MoSCoW (Must have = **M**, should have = **S**, could have = **C**, won’t have this time = **W**)

Dimensions	Interviews	Literature review	App review
Features	• Searchable **(M)** • Links to genetic services/ • webpages **(M)** • Link to WhatsApp or other pregnancy related Apps e.g. Bounty **(C)**	• Preloaded information videos **(M)** • Reporting features **(M)** • Store data on device **(M)** • Search tool (e.g. lookup/find words/terms in text) **(C)** • Alerts/notifications **(C)** • Service planning **(W)** • Links to health record **(W)**	• Should be able to work offline **(M)** • User instructions. Record user preferences. Guide **(M)** • Share data. Display e.g. graphs. Communicating with provider/social support **(W)** • Alert/remind. Evaluate **(W)** • To intervene **(W)**
Accessibility	• Urdu, English & Punjabi all mentioned with subtitles **(M)** • Large enough writing to prevent zooming in **(M)** • Expectations that any referral could be conducted in Punjabi/Urdu **(M)**	• Being able to zoom in/out to read text **(M)** • Plethora of colour-enhanced features **(M)** • Use of primary languages **(M)** • Works on different display resolutions/screen sizes **(M)**	• Free to download but could have in-app purchases **(M)** • Available in Apple and Google Play **(M)** • High reading score e.g. 77/100. Low reading age e.g. US grade 8 **(M)** • Available on NHS library **(W)**
Design	• Drop down menu or multiple choice to reduce text entry (if not proficient in English) **(M)** • Icons for more information **(M)** • Patient stories and clinician videos and audio **(M)** • Images & cartoons to break up the writing **(M)** • Easily navigate to home page **(M)** • Short bulleted text **(M)** • Pictures & diagrams (though not of children with disabilities - might be off-putting **(M)** • Pictures of health professionals who looked like them e.g. head scarf **(M)**	• Input validation **(M)** • Use of colour coded information **(S)** • Offline mode **(C)** • Road map/framework (e.g. bread crumbs) **(C)** • Animations to aid understanding **(C)** • Checklist to show completed areas **(C)**	• 23% of apps recorded data **(S)** • Icon and name related to genetics 68% **(S)**
Content	• Analogies of baking, sport, prayer beads to explain missing or faulty genes **(M)** • Explanation of risk **(M)** • Cousin marriage and blood relative **(M)** • Explanation of inheritance and risk **(M)** • Descriptions of some common conditions **(M)** • Links to further support - reducing the fear of a genetic condition e.g. charities or web pages describing conditions **(M)** • Explanation of gene pool or new blood **(S)** • Link to religion make clear having a genetic test is halal **(W)**	• Use of Pictures and videos rather than lots to read **(M)** • Usability of the app in illiterate people **(M)** • The content display format gets adjusted as per phone model making it easy to read for the elderly and even less adept smartphone users **(M)** • Patient stories **(M)** • Visualisations **(M)** • Personalisation **(S)**	• Must educate/inform − 100% of reviewed apps did that **(M)** •Analyse DNA (32% of apps)**(W)** • Assist diagnoses 18% **(W)**
Quality	• Links to credible sources e.g. charities, medical journals and genetic testing centres **(M)** • NHS logo & branding **(M)** • what to expect from a referral to a genetic service **(W)** • Link with a professional for further support **(W)**	• User friendly **(M)** • Interoperability **(S)** •Modular architecture-Interoperability **(S)**	• MARS > 3 (highest was 4.1). High engagement e.g. funny, functionality e.g. ease of use and intuitive, aesthetically pleasing, high info quality **(M)** •Affiliated with professional body **(M)**
Security	• Privacy settings e.g. via facial or thumb print recognition **(M)** • Icon or title in App store not obvious link to genetics - pairs of jeans suggested **(M)** • Explanation of data collection policy and what is being collected **(W)**	• Data encryption **(M)** • Passwords/pins **(M)**	• Privacy policy (with clear explanation of third party sharing if happening) and log in if collecting/storing data **(M)**

**Fig. 2:**
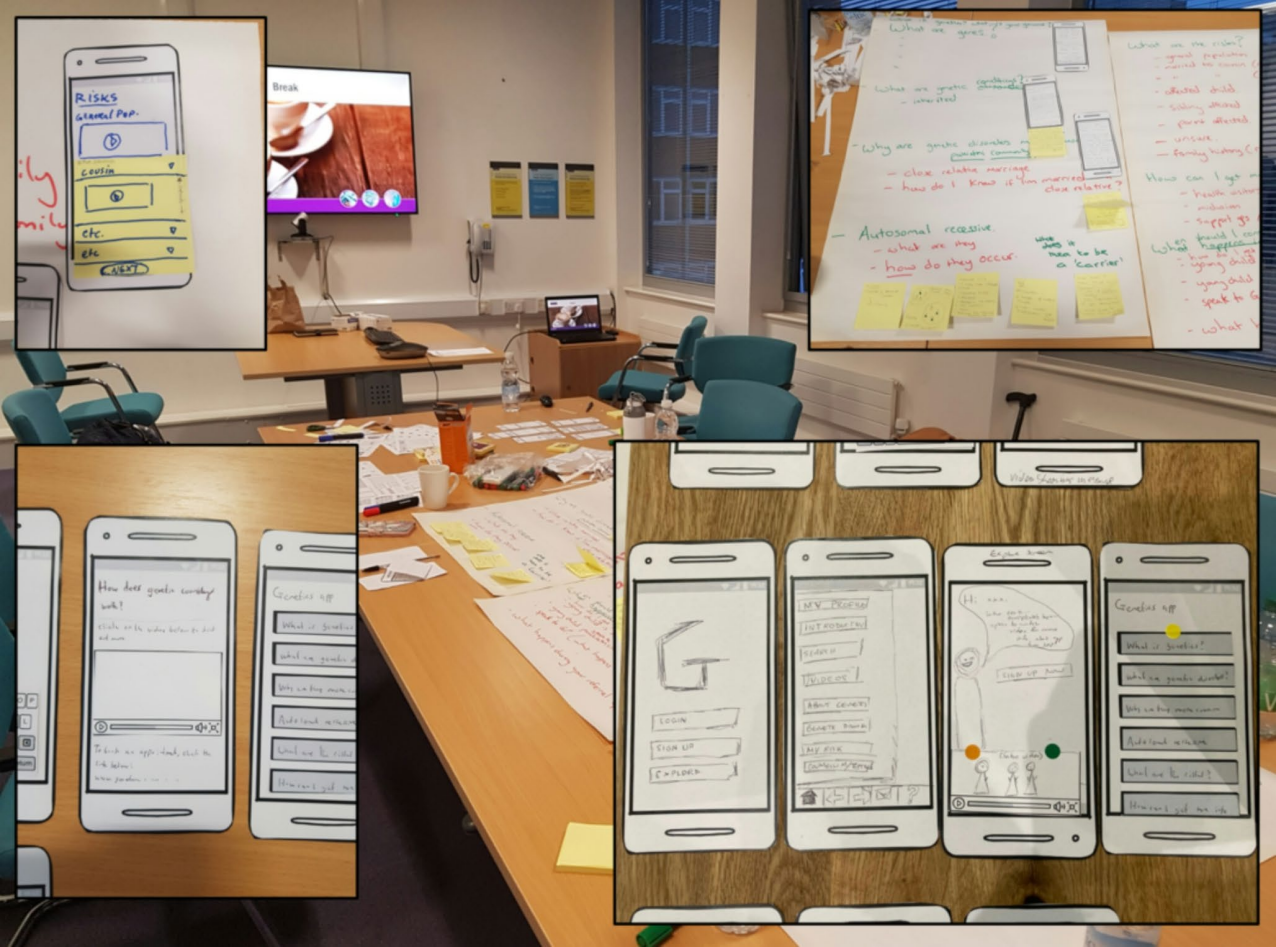
Various paper prototypes generated during the initial design session with subject matter experts and ordered by narrative

Initial paper prototypes were then developed through a series of workshops with subject matter experts during the COVID-19 pandemic (Fig. 2), including a colleague with expertise in design thinking. Based on the interviews with participants, the group first established the content that the app would need to cover, including:

- What are genes?
- What are genetic conditions?
- Why are genetic conditions more common in the Pakistani community?
- What does autosomal recessive mean?
- What are the risks?
- How can I get more information?
- When should I consider a genetic test?
- What is genetic testing?
- What happens when you get your results?

The findings discussed by the group following the paper prototyping approach are presented in Table 3.

**Table 3:** Itemised content and format used in the app

Content Type	Content Covered
Text	● Why is genetics important to me and my family? ● What are genes? ● What are genetic disorders? ● Close marriage ● What is a genetic test? ● Getting more information ● Glossary
Animations	● What are chromosomes? ● What are the risks? ● What does it mean to be a carrier? ● What happens at genetic testing? ● What makes your genome?
Video	● Genetics clinic consultation ● Genetic counsellor describing patient journey through genetic testing ● Genetic counsellor describing the process of receiving a genetic test result ● Overview of the app and purpose

Animations were designed by a professional design company (Niftyfox), following extensive storyboarding of the concepts with the project team. The storyboards were reviewed by two of the original participants during a face-to-face feedback session with the project team and the animation company. Areas that were flagged included skin colour of animated characters, clothing, analogies used and also representation of both male and female in each animation to ensure it was clear that a recessive genetic condition was inherited from both parents. Further iterations of the storyboards were made according to the feedback received during this session.

Video content was produced by the in-house media services team at The University of Manchester, scripts were written by the project team, including a genetic counsellor to ensure their authenticity. Actors were used to portray the clinicians and patients in the videos and were purposefully selected to ensure they had the right ethnicity. One of the actors could also speak Urdu, which enabled us to capture the voiceover of the videos in Urdu.

**Fig. 3:**
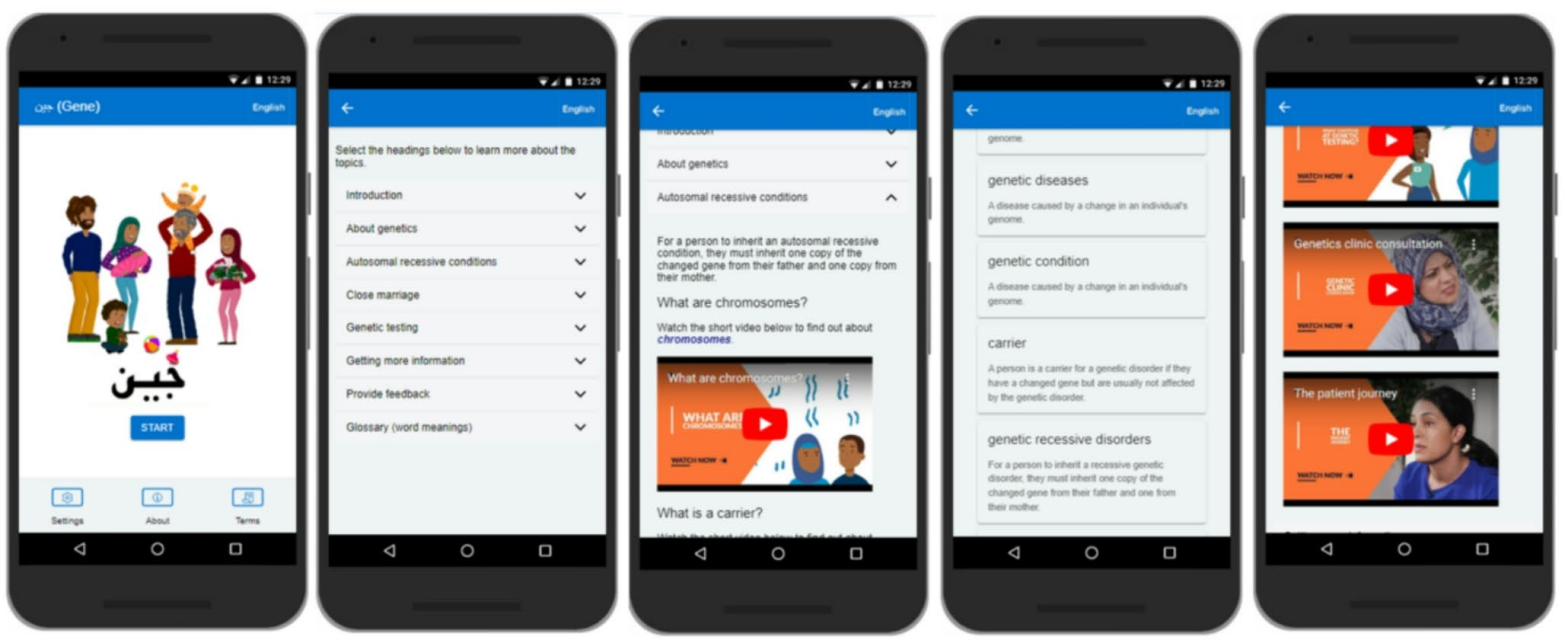
Screenshots of the application showing (from left to right) the home screen, main menu, animation content, glossary and videos of mock clinic consultations

Prototypes were then further developed into a high-fidelity digital prototype in the form of an Ionic mobile app. The separately designed animations and video content were also added to the app (see Fig. 3).

Two female participants reviewed the content and proposed some changes which were discussed by the team and prioritised using the MoSCoW method (see Table 4).

**Table 4:** Proposed changes and issues Raised with MoSCoW prioritisation

Proposed change / issue	Complexity	Comments	MoSCoW
Replace landing page/screen image as it looks like an Indian/Pakistani wedding	Easy	Consider using an image of a family	S
Info in videos is brief - just clips of one-minute videos	Hard	Requires new video content	W
Not enough information in some of the videos for someone learning more about this	Hard	Requires new video content	W
Should be NHS branded to increase trust	Hard	We are aiming for this in the longer term	W
Add different colours to the writing on the app	Easy	Would not recommend for dyslexic readers. Good design guidance also mentions using no more than 3–4	-
A lot of writing - too wordy. Can this be reduced more?	Medium	Would make this more accessible but is hard to do while still retaining meaning	S
Change the written language options to use icons of the UK and Pakistan flags	Medium	We had this initially but removed it due to previous feedback about excluding other communities	-
Add the language selection choice to a popup when first access app	Medium	This would maximise the utility of the language options	M
Change collapsible/expandable menu to coloured squares (menu buttons)	Medium	At present all information is on a single app page in collapsable sections. Changing this to menu buttons would involve creating a new app page and associated resource files for each section	C
Don’t use the word ‘glossary’ as it wasn’t clear what this means, maybe ‘meaning of words’	Easy	They felt that only people who had completed a degree would understand this word and a different word or phrase should be used	M
Change the typeface “it’s not cool”	Easy	Currently uses a web safe font recommended by the NHS style guidelines.	W
Video implies the genetic test checks all the DNA (e.g. like a ancestral genetic testing service) rather than just looking at specific genes associated with suspected conditions	Hard	Requires new video content or editing existing video	S
Video of people talking where they say “I’m worried about the genetics” and the response is “there is nothing to worry about” - needs to be explained this is about the appointment and not that there is nothing to worry about having a genetic condition	Hard	Requires new video content or editing existing video	S
The question and answer section was too binary considering there can be many possible answers	Medium / hard	These represent one such answer and not all situations. Not sure how to address this. Add a statement that this is one possible answer?	C
Interactive question section - not clear that it’s not asking several questions in one go (due to title, subtitle and question)	Easy	We can make this clearer by altering text	M

Note: - means this will not be addressed

### Phase 4. In-person feedback session and app refinement

Five community members tested the app for usability and acceptability. We also explored their responses to the data collection methods.

#### Usability

The mean SUS score was 87 (SD: 10.95; range: 72.5–97.5), indicating excellent usability. No challenges in using the app were observed. However, three participants initially chose to watch the videos together and read the written content individually.

Participants found moving between screens easy (Mean: 4.6; SD: 0.55; range 4–5). They also found it easy to learn to use the app (Mean: 4.4; SD: 0.89; range 3–5). They explained:

*It was easy to use the app. The app flowed from one section to the next. (P1)*

*Everyone knows how to use a device and how to use an app*,* as long as they know how to read the language. It was easy to navigate through the app. (P5)*

#### Acceptability

Acceptability data is presented in Table 5. The written information, animations, videos and content were acceptable to participants. Survey scores were lower for the items *‘the written information was comprehensive and concise’* and ‘*the content could be improved’* indicating that the app could be improved. Participants suggested more use of videos, simplifying the language used (avoiding jargon and medical terms), translating the app into other languages (especially Urdu) and adding other content, like links to support groups and written testimonials from patients who have used a genetics service. Participants also wanted more information on what to do next, including charities they could link with or a ‘communication box’ where they could send a message to someone who can direct them to services or resources.

Importantly, participants tended to trust the information in the app (Mean score 4), citing reasons such as that the app is science-based. One participant responded that she was ‘unsure’ about whether the information in the app was trustworthy. She explained that she would always get a second opinion with anything health/medical related. Two others said they trusted the information as the researchers had made an effort to travel to the community and had research experience on the topic. There was discussion around the idea that they might trust the information more if it was in Urdu, as they could understand this better. Participants also said that testimonials would be helpful for building trust in genetics services.

**Table 5:** Acceptability of the gene app

	Mean (SD; range)	Qualitative feedback	Improvements
**Written information**			
The text was easy to understand	4 (1; 3–5)	The written information is informative. Easy to understand in the keyword/glossary section. More YouTube videos. Different languages. Could improve if have in different languages. Can use simple words instead of different word.	● More videos ● Different languages ● Simplify words used
The written information was comprehensive and concise	3.8 (1.10; 3–5)		
**Animations and videos**			
The animations and videos were visually appealing	4.4 (0.55; 4–5)	The Genome video I will need to watch a couple of times to understand. The Genome video to be simplified.	● Simplify Genome video
The animations and videos were clear to understand	4.4 (0.55; 4–5)		
**Layout and content**			
The layout of the content was appropriate	4.6 (0.55; 4–5)	Support group links. Written testimonials. More videos and different languages - Urdu.	● Add links to support groups ● Add written testimonials ● More videos ● Different languages (Urdu)
The content could be improved*	3 (1.22; 1–4)		
**Trust**			
I trust the information in the app.	4 (1; 3–5)	It is evident on the app that it is researched and science based. The people who made it have research experience.	
**Other feedback**	Different languages- Urdu. More videos- more understanding. More simple words, rather than jargon or medical terms. I was told not to research anything about genetics, as I will learn everything during the session. I can assure you I have learnt a whole lot more than I imagined. More than I did many years ago in science lessons.		● Different languages (Urdu) ● More videos ● Use simple words

Note: *Lower score reflects a more positive response as the item is framed negatively

#### Responses to the data collection methods

In the other phases, community members responded well to the online interviews. In Phase 4, they also tended to generally respond well to the SUS and bespoke questionnaire items, with all items responded to. However, three participants in Phase 4 sought clarification on what ‘integration’ and ‘inconsistencies’ meant (SUS items 5 and 6 Brooke 1996). Participants also tended to struggle more with the open-ended written questions, with many choosing not to answer them. Some participants accepted help in responding to the open-ended questions, opting to have a researcher transcribe some of their responses. This may have reflected language barriers, as the questions were written in English.

## Discussion

### Community-based co-design

Within this project, we have co-designed and co-created a prototype of the smartphone app Gene with the British Pakistani community, deployed on both Android and iOS devices. The approach was democratic, creating opportunities for British Pakistani community members to be involved in the creation process, regardless of their previous design and development expertise (Carr et al. 2009).

A strength of this approach has been the engagement of the community with the research project, facilitated by building trust and respect by NK working in a public health outreach role for several years before the project began. This enabled NK to draw on existing connections with community groups for participant recruitment, who in turn facilitated recruitment from voluntary community groups such as Homestart which support Pakistani families within the local communities. Similar conclusions about building trust for inclusive research in South Asian communities have been made by (Ali et al. 2024). The authors emphasise that researchers who share the ethnic background of the participants and leveraging the support of key groups in South Asian communities are essential to support digital health research in the community (Ali et al. 2024). Time must also be invested to source and develop these networks to build trust and respect before the commencement of any such research study.

In some of the interviews conducted in Phase 1, participants expressed concern that it is often perceived by males that genetics is passed down the female line in Pakistani communities. Other research has also reported similar misconceptions, such as that people inherit more biological material (e.g., cells or genes) from one parent than the other (Shaw and Hurst 2008; Klitzman 2010). This finding highlights that misconceptions about genetic inheritance can shape health beliefs and potentially contribute to gendered perceptions of responsibility for genetic conditions. Addressing such misconceptions (as we have done in our animations) is crucial for promoting informed decision-making, reducing stigma, and ensuring equitable participation of both partners in discussions about genetic risk. This also highlights the importance of culturally sensitive genetics education materials that challenge inaccurate beliefs and present genetic information in an accessible manner.

The lack of male participation during the initial interviews and feedback stages of development is important to acknowledge. Males in Pakistani families can often drive decision-making, and so this is an important voice that needs to influence the future design of the app to ensure its use by both males and females, and that content is applicable and acceptable by both sets of users. During Phase 4, female participants suggested to the research team that it would be helpful to get male relatives involved in providing feedback and that they often did not engage in discussions related to genetics until they had an affected child.

The role of older family members was also an important consideration for both the design and usage of the app. This is because the Pakistani family-centred model of decision-making is valued, with family or medical doctors often making medical decisions rather than the patient alone (Moazam 2000). This is in part due to family obligations and harmonious living within multi-generational households (Moazam 2000). It was clear during the interviews in Phase 1 and feedback session in Phase 4 that older family members were central to the decision-making process. Participants discussed that whilst they might not use the app themselves, younger family members might show it to them on their phones, and this approach was also observed during feedback sessions with family members choosing to view and discuss the content together.

Feedback from participants has helped to ensure that the analogies used are culturally appropriate. Analogies can be useful to explain medical information (Hildenbrand and Perrault 2022) or to influence healthcare decision-making (e.g., willingness to change treatments) (Gasteiger et al. 2020). However, different cultures conceptualise health in different ways, which can result in miscommunication (Magaña 2019). Others have also discussed using culturally relevant analogies. For example, Prinjha et al. (Prinjha et al. 2020) found that participants involved in a study exploring mHealth text messaging to support type 2 diabetes medication adherence were interested in culturally relevant content, such as herbs and spices they might use in their food and religious fasting. In our work, we discussed analogies related to prayer beads and the baking of foods such as chapatis; however, content could be personalised in the future according to analogies that might resonate more with males and older family members.

### Language

All content within the app including animations, videos and written content was translated into Urdu. Other languages were also suggested during the Phase 1 interviews such as Punjabi. There were challenges with creating content in Urdu in addition to English as there are some words and phrases that might be used to describe genetics, that do not translate directly and thus need describing in more detail as a concept. Practically this led to challenges during animation as the animation had to be slowed down to accommodate these lengthier explanations. There were other practical considerations for creating the videos; though NK and one of the actors both spoke Urdu, our media team did not - which meant post-editing was more challenging.

### Strengths and limitations

Throughout the four phases, we adhered to the principles of co-design: collaborating with stakeholders (participation), iterative development, collective ownership and having a practical focus (outcomes and intent) (Donetto et al. 2014). We also used differing methods of obtaining feedback, including observations, interviews and surveys. Participants’ prior interactions with genetics services were also varied, ranging from none to long-term involvement over decades. These perspectives were useful in capturing different use cases that could be modelled from those with no prior knowledge of genetic services through to participants with many years of experience and the benefit of and their reflections regarding what information would have been helpful to them during their journey with genetic services.

As discussed earlier, a limitation was the lack of representation of the male perspective and of older community members, who may have different insights into the topic and are key decision-makers in the community. The evaluation was also limited to English-speakers highlighting the need for further testing of the Urdu translations. In the future, testing with a larger and more diverse sample would also be beneficial. Another limitation is that documentation (e.g., patient information sheets, consent forms and surveys) were not proofread for readability by public representatives prior to use. This would have minimised some minor issues in understanding all items within the SUS survey.

## Conclusion

Four phases were conducted to co-design and co-create a new accessible and culturally sensitive genetics information resource with and for the British Pakistani community. Preliminary acceptability and usability testing of the Gene app by community members found that it was deemed acceptable and rated excellent in usability. The collaborative nature of the approach, especially partnering with a trusted genetics expert in the community, was integral to the success of the project. Future testing of the app is needed, focussing on males and older community members who also play a central role in medical decision-making.

## Electronic supplementary material

Below is the link to the electronic supplementary material.

Supplementary Material 1

## Data Availability

Participants did not give consent for their data to be shared, so supporting data is not available.
