# Supplementary material for: Co-designing ‘gene’, a smartphone app for genetics education and empowerment with and for the British Pakistani community: a methodological summary of the GENE-Ed project
Source: J Community Genet. 2025 Apr 12;16(3):267–82. doi: 10.1007/s12687-025-00789-0 (PMC12202270; doi:10.1007/s12687-025-00789-0)
Supplement: Supplementary file 1 — Supplementary Material 1 [file 12687_2025_789_MOESM1_ESM.docx]

**SUPPLEMENTARY FILE**

**Appendix 1. Interview script for Phase 1**

1. Can you describe your ethnic background, age and your highest educational attainment to date and employment status? Have you ever been a patient in the genetics service?
2. If you had concerns around genetics ( for eg or some concern about a health problems or disabilities in the family) /cousin marriage and you wanted to access information: What would you do? Who would you ask?
3. Would you use the internet/(e.g., Google)? What words would you put into an internet search? Any other words you might search for? (obtain all words they may enter)
4. What sorts of questions would you want answering? What information would you be looking for? Ascertain their specific questions/start generic
5. Have you heard of the terms consanguinity (or/both) cousin marriage? What do the words mean to you? (ascertain connotations associated help us decipher what terminology is acceptable etc)
   Consanguinity: anybody that’s related by blood. What are your preferred terms/words? Are there any other words you are aware of with relation to this? Also use the term blood relatives.
6. What does genetics mean to you? What comes to mind with the word genetic? How would you explain/describe genetics? eg its what makes us individual and what we inherit from our parents and our instructional manual for what makes us , us.
7. If you had a child with genetic condition /or family history of genetic condition what specific information would you would want to access/be looking for: ask them what they would want to find out as an open questions. Use following prompts to enable us to obtain information we need: What are genes? Gene changes and how they are passed on in families? How gene changes cause conditions. Risks to future children. How do I stop it being passed on? What can I do to prevent it happening again? How can I access services? What services are available?
8. Do you use a smartphone or other device such as a tablet? If yes: Do you regularly use Apps on this device and if so which ones? What do you like about the Apps that you use? Have you already used an App or specific website to find out more about genetics? Would you use an App to find out more information about genetics?
9. If you were to use an App to find out more information about genetics, how would you like the content to be presented? For example, short videos, patient stories, animations, pictures, written descriptions
10. How would you use the App? For example: On your own, with other family members, with your partner, with a healthcare professional or with somebody else?
11. How would you like to App to be presented?
12. Do you think an App is the best way to deliver this information?
13. Would the App need to be integrated into a wider service or could be used standalone?
14. Is there anything else you would like to add?

**Appendix 2. Surveys used to evaluate the app in Phase 4.**

**App evaluation survey**

In this survey we would like to know what you thought about the جین (Gene) app.

Please select the answer that best expresses how you feel about each statement and write more feedback in the boxes.

|  | Strongly Disagree | Somewhat Disagree | Neutral | Somewhat Agree | Strongly Agree |
| --- | --- | --- | --- | --- | --- |
| 1. The text was easy to understand. |  |  |  |  |  |
| 1. The written information was comprehensive and concise. |  |  |  |  |  |

Why did you think this? How would you improve the written information?

|  |
| --- |

|  | Strongly Disagree | Somewhat Disagree | Neutral | Somewhat Agree | Strongly Agree |
| --- | --- | --- | --- | --- | --- |
| 3. The animations and videos were visually appealing. |  |  |  |  |  |
| 4. The animations and videos were clear to understand. |  |  |  |  |  |

Why did you think this? How would you improve the animations and videos?

|  |
| --- |

|  | Strongly Disagree | Somewhat Disagree | Neutral | Somewhat Agree | Strongly Agree |
| --- | --- | --- | --- | --- | --- |
| 5. The layout of the content was appropriate. |  |  |  |  |  |
| 6. The content could be improved. |  |  |  |  |  |

Why did you think this? How would you improve the content in the app?

|  |
| --- |

|  | Strongly Disagree | Somewhat Disagree | Neutral | Somewhat Agree | Strongly Agree |
| --- | --- | --- | --- | --- | --- |
| 7. I trust the information in the app. |  |  |  |  |  |

Would you trust the information in the app? Why or why not?

|  |
| --- |

**USABILITY QUESTIONS**

|  | Strongly Disagree | Somewhat Disagree | Neutral | Somewhat Agree | Strongly Agree |
| --- | --- | --- | --- | --- | --- |
| 8. Moving between the screens was easy. |  |  |  |  |  |
| 9. It was easy to learn to use the app. |  |  |  |  |  |

How easy or challenging was it to use the app?

|  |
| --- |

**SYSTEM USABILITY SCALE**

Please select the answer that best expresses how you feel about each statement, after having used the app.

|  | Strongly Disagree | Somewhat Disagree | Neutral | Somewhat Agree | Strongly Agree |
| --- | --- | --- | --- | --- | --- |
| 1. I think I would like to use this app frequently. |  |  |  |  |  |
| 1. I found the app unnecessarily complex. |  |  |  |  |  |
| 1. I thought the app was easy to use. |  |  |  |  |  |
| 1. I think that I would need the support of a technical person to be able to use this app. |  |  |  |  |  |
| 1. I found the various functions in this app were well integrated. |  |  |  |  |  |
| 1. I thought there was too much inconsistency in this app. |  |  |  |  |  |
| 1. I would imagine that most people would learn to use this app very quickly. |  |  |  |  |  |
| 1. I found the app very cumbersome (inconvenient or clumsy) to use. |  |  |  |  |  |
| 1. I felt very confident using the app. |  |  |  |  |  |
| 1. I needed to learn a lot of things before I could get going with this app. |  |  |  |  |  |

**OPTIONAL FEEDBACK**

**You can skip this page, but if you would like to give us more feedback, please write in the boxes below.**

What did you think of the app? How would you improve it?

|  |
| --- |

Is there anything else you want us to know? (Other feedback)

|  |
| --- |

**Thank you so much for your time today! We really appreciate your feedback.**

**Please give this form back to the researchers.**
